# Quantification of Nicotine and Cotinine in Plasma, Saliva, and Urine by HPLC Method in Chewing Tobacco Users

**DOI:** 10.31557/APJCP.2019.20.12.3617

**Published:** 2019

**Authors:** Fareeda Begum Shaik, G Nagajothi, K Swarnalatha, C Suresh Kumar, Narendra Maddu

**Affiliations:** 1 *Department of Biochemistry, Sri Krishnadevaraya University, Ananthapuramu, Andhra Pradesh, *; 2 *Department of Corporate Secretary ship, Queen Mary’s College (Autonomous), Chennai, Tamil Nadu, India. *

**Keywords:** Nicotine, cotinine, plasma, saliva, Urine

## Abstract

**Background:**

Nicotine acts as major alkaloid of all tobacco products including smokeless tobacco (SLT) forms. The mode of SLT consumption is in the form of chewing under the cheek or lip and induced biochemical alterations in the plasma, saliva, and urine.

**Materials and Methods:**

The smokeless tobacco products like Raja or blue bull tobacco brands are widely consumed by human male volunteers under the age of 18-30 years for the period of 3 years consisting of 30g per day. The concentrations of nicotine and cotinine in samples of plasma, saliva, and urine are quantified by the method of HPLC. The remaining variables of plasma are evaluated by auto analyzer and spectrophotometric methods.

**Results:**

The analysis of results presented that significant increase in the levels of nicotine and cotinine in plasma, saliva, and urine of chewing tobacco users. The lipid profile (Cholesterol, triglycerides, HDL-C, and LDL-C), liver marker enzymes (SGOT, SGPT, and ALP), kidney markers (Creatinine, urea, and uric acid), glucose, and the remaining variables are present within normal range observed in SLT users. The lipid peroxidation (LPO), nitric oxide (NO) (NO_2_ and NO_3_), protein carbonyls (PCO), and peroxynitrites (ONOO-) are reported to be higher levels in the plasma of experimental subjects in comparison with normal controls. The various brands of tobacco varieties (Raja, madhu chhap, hans chhap, miraj, badshah, blue bull, and swagat gold tobacco) are presented.

**Conclusion:**

The chewing tobacco users exhibited greater amounts of nicotine and cotinine are at risk of cardiovascular due to nicotine has cardiovascular effects, and oral cancer disease complications in the future for chronic consumption of smokeless tobacco products due to the presence of carcinogens of tobacco-specific N-nitrosamines.

## Introduction

The oral consumption habit of smokeless or chewing tobacco is alarming a global threat and toxicity in the form of negative health consequences to human health (Biswas et al., 2015). The smokeless tobacco has been classified as a group 1 carcinogen to humans was reported by the working group of International Agency for Research on Cancer (Warnakulasuriya and Straif, 2018). The increasing awareness, pictorial warning labels, and anti-tobacco campaigns about smokeless tobacco products results in the decrease of 34.1% of consumers was revealed by Global Adult Tobacco Survey-2016-17 in the India (Shah et al., 2018). The total 356 million people are the habitual users of smokeless tobacco across the globe (Mehrotra et al., 2019). The greater satisfaction and psychological reward was highly observed in regular snus users compared to occasional users (Zandonai et al., 2018). The state wide tobacco control and prevention efforts that address the different types of smokeless tobacco products consumption among adults (Hu et al., 2019).

There is correlation between smokeless tobacco usage and oral mucosal disorders (Hallikeri et al., 2018). The association was stronger between tobacco use and young adults with lower educational attainment and below the poverty level (Lienemann et al., 2019). The chronic consumption of smokeless tobacco is associated with the development of oral potentially malignant disorders and cancers of oral cavity, oesophagus and pancreas have been reported (Gupta et al., 2018). The use of smokeless tobacco (chewing tobacco, snus, snuff, and dissolvable tobacco) is higher among adults under age of 18 and older (Wang et al., 2018). The consumption of smokeless tobacco extract (STE) is growing rapidly, and it has been implicated in several human diseases including diabetes, inflammation and a number of types of cancer (Li et al., 2018). The factors of educational inequalities in SLT use were higher in urban areas of India and in rural areas of Bangladesh, whereas the wealth inequalities in SLT use were higher in urban areas of both the countries (Bandyopadhyay and Irfan, 2019). 

It is a misleading advertising by tobacco companies may be responsible for the increase in the SLT prevalence, which is as harmful as smoking (Suliankatchi et al., 2019). Use of smokeless tobacco leads to potentially preventable, global morbidity and mortality diseases. The World Health Organization needs to consider incorporating regulation of the smokeless tobacco into its Framework Convention for Tobacco Control (Siddiqui et al., 2015). The purpose of the present study is to explore the levels of nicotine and cotinine in plasma, saliva, and urine and biochemical alterations in plasma of chewing tobacco users.

## Materials and Methods

*Study subjects and Data collection*

Sixty human male volunteers were selected and each group consisted of thirty volunteers, aged between 18-30 years residing in Ananthapuramu town, Andhra Pradesh taking local diet. The baseline information for the category of SLT users was that individuals used SLT products (Blue bull, and raja brands of tobacco) habitually, at least > 4 times per day consists of 30 g during the last 3 years. Socio-demographic information was collected by an interviewer with the information on age, educational qualification, marital status, income, and occupational status. The inclusion criteria are the habitual use of only SLT packets by the users, and choose the unmarried and low economic status people. In the present study all volunteers were free from any chronic disease, with no smoking habit or alcohol drinking. All experiments were performed in accordance with the approved guidelines and regulations of the Ethical Committee (No.25/1/2019-AWD).

*Group I- Controls*

*Group II-Chewing tobacco users*

*Collection of blood and Sample analysis*

Blood samples from overnight fasted subjects were used for the study. Blood samples, drawn from human male volunteers by vein puncture between 7 and 10 AM into heparinized test tubes, were used immediately for plasma analysis. Most of the plasma clinical parameters were estimated by auto analyzer kit methods. Plasma lipid peroxidation was analyzed by the method of Buege and Aust, (1978), iron by Ramsay (1958), total amino acids by Moore and Stein, (1948), glycolipids by Roughan and Batt (1968), total phospholipids by Connerty et al., (1961), peroxynitrites by Beckman et al., (1992), protein carbonyls by Levine et al., (1990), nitric oxide (NO_2_ and NO_3_) by the method of Sastry et al., (2002). 

*Saliva-urine collection and Analysis*

Five millilitres of unstimulated whole salivary samples were obtained by expectoration, in the absence of chewing movements, in dry plastic vials with the test subject sitting in a relaxed position. The collected saliva samples were centrifuged at 3,000 rpm for 10 min. The supernatants were stored at -70°C until further analysis. Two millilitres of urine samples in the morning were collected in a sterile flask covered with aluminium foil to keep out stray light and processed within 2 h of the collection. The collected samples were centrifuged at 3,000 rpm for 10 min for further analysis.

*HPLC*

HPLC system (Shimadzu, Japan) is equipped with a binary gradient system with variable UV/VIS detector (SPD-20A) and Rheodyne injector with a 20 µL loop and LC-20AD pumps and integrator. Reversed phase chromatographic analysis was performed in isocratic condition using C18 reverse phase column (5 µ) at 37°C. 

*HPLC operating conditions*

Resolution of peaks was performed with the mobile phase consisting of a mixture of 0.272 g of KH_2_PO_4_, 0.184 g of sodium n-heptane sulfonate, 820 mL of water (HPLC-grade), and 180 mL of methanol (HPLC grade). The pH of the mobile phase was adjusted by drop wise addition of ortho phosphoric acid (pH=3.2). The flow rate used was 1.0 mL/min, and the wavelength was fixed at 256 nm for nicotine and 262 nm for cotinine as per the modified method (Massadeh et al., 2009). Nicotine and cotinine at the concentration of 20µM/mL were used as standards.

*Sample analysis for HPLC*

Plasma sample analysis was processed by the modified method (Massadeh et al., 2009). An aliquot of 0.1 mL plasma was placed into a glass test tube was alkalinized with 20 µl of 5.0M NaOH, and vortex mixed at 2800 rpm for 30s. An equal amount of dichloromethane-diethyl ether (1:1 v/v) was used for one-step single extraction, and then vortex mixed at 2800 rpm for 2 min. The organic layer, after being centrifuged at 3500 rpm for 3 min, was transferred to a new glass tube containing 4 µL of 0.25M HCl. The organic layer, centrifuged at 3,500 rpm for 3 min, was transferred to a new glass tube containing 4 µL of 0.25M HCl. The organic phase was then evaporated under a stream of nitrogen at 35ºC until dryness and reconstituted in 50 µL of mobile phase. An aliquot of 20µL was injected into HPLC for analysis.

*Statistical data analysis*

All the quantitative data are expressed as mean ± SEM and Students t-test was used to determine the significance of the parameters between the groups. The Pearson correlation coefficient analyzed using Graph Pad Prism version 6.01 for Windows. A P value less than 0.05 was considered statistically signiﬁcant.

## Results

*HPLC chromatograms of nicotine and cotinine in plasma, saliva, and urine*

**Figure 1:**
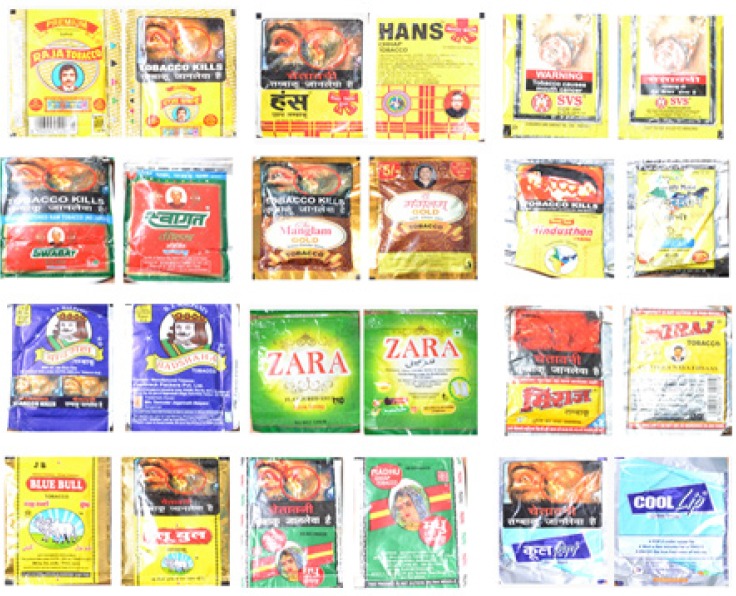
Images of Raja, Hans Chhap, SVS Madras Snuff, and Swagat Gold Tobacco Brands

The photographs of various different chewing tobacco products like Raja, Hans chhap, SVS madras snuff, Swagat gold, Hindusthan khaini, Manglam gold, Zara snus, Badshaha, Miraj, Cool lip filter tabaq, Blue bull, and Madhu chhap chewing tobacco are presented. The pictorial health warning labels are in the form of Tobacco Kills or Tobacco causes Mouth Cancer (Figure 1). 

Data (Figure 2a) indicated the range of retention time of standard nicotine is 5.1-6.5 min and appeared a chromatogram peak at 6.38 min. The range of retention time of standard cotinine is 3.6-4.2 min and manifest that a chromatogram peak at 4.01 min. The normal control group had no nicotine intake and tobacco exposure. Minimal concentrations of nicotine and cotinine levels are noticed in control group due to environmental tobacco exposure and some food constituents. There are no peaks declared in chromatograms of plasma, saliva, and urine in normal healthy controls at the retention of 4.01 and 6.38 min of nicotine and cotinine respectively. Chewing tobacco consumers reported that narrow nicotine chromatogram peak at retention time of 5.84 min and cotinine peak at 3.68 min in the plasma. In this group II, large amount of nicotine is metabolized into cotinine. In the saliva sample, nicotine disclosed a chromatogram peak at 6.23 min retention time and cotinine exhibited that chromatogram peak at 3.86 min in group II consumers. The chromatograms of nicotine and cotinine at 5.12 min and 3.84 min respectively in the urine of smokeless tobacco consumers (Figure 2b). Biochemical profile in plasma

The decreased levels of HDL-C, and increased concentrations of lipoproteins (VLDL-C, LDL-C), kidney markers (Creatinine, urea, and uric acid), liver marker enzymes (SGOT, SGPT, and ALP), cardiovascular markers (Cholesterol and triglycerides), and phospholipids are perceived in the plasma of chewing tobacco users. The mean values of HDL-C and total cholesterol have been visible a statistically significant difference. The levels of reactive oxygen nitrogen species include nitric oxide (NO_2_ and NO_3_), protein carbonyls, peroxynitrites, and malondialdehyde are significantly higher in smokeless tobacco users compared to normal subjects (Figure 3a, 3b, and 3c). 

*Concentrations of nicotine and cotinine in plasma, saliva, and urine*

Smokeless tobacco users evinced that significantly increased levels of nicotine and cotinine concentrations in plasma, saliva, and urine compared to group I controls. The correlation plots of plasma nicotine and cotinine with salivary and urine nicotine and cotinine (Figure 4). The distribution of nicotine and cotinine concentrations in urine, plasma, and saliva are presented (Figure 5a, 5b, and 5c).

**Figure 2a.**
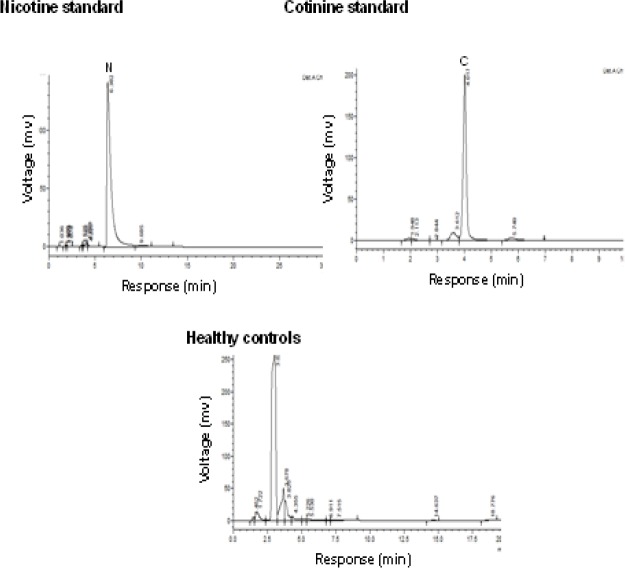
HPLC Chromatograms of Nicotine, Cotinine Standards, and Healthy Controls

**Figure 2b:**
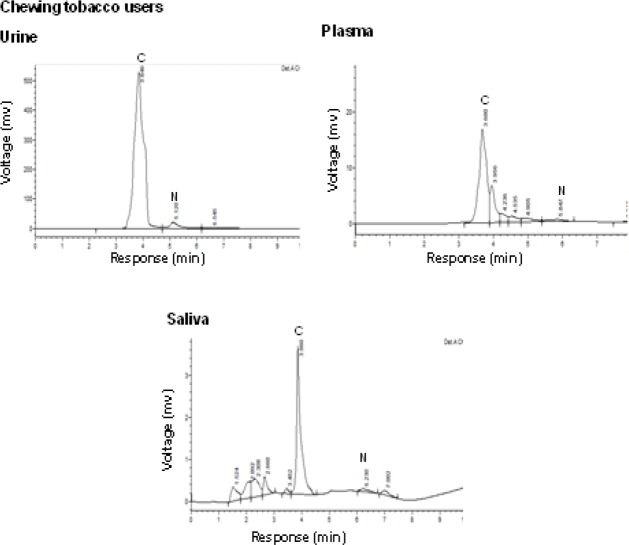
HPLC Chromatograms of Smokeless Tobacco Users

**Figure 3a:**
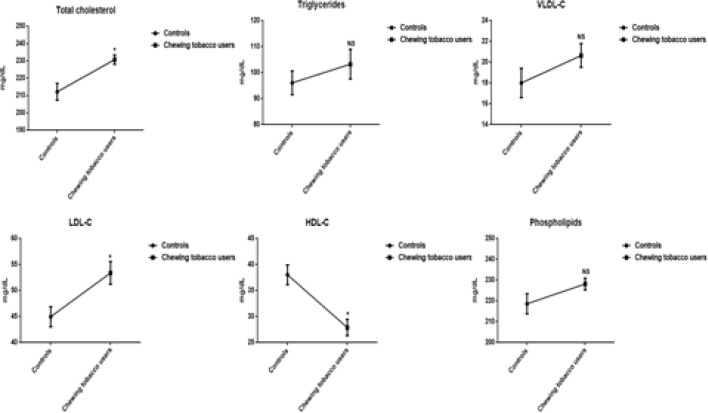
Lipid Profile in Plasma of Chewing Tobacco Users. Note: VLDL-C-Very low density lipoprotein cholesterol; LDL-C-Low density lipoprotein cholesterol; HDL-C-High density lipoprotein cholesterol

**Figure 3b:**
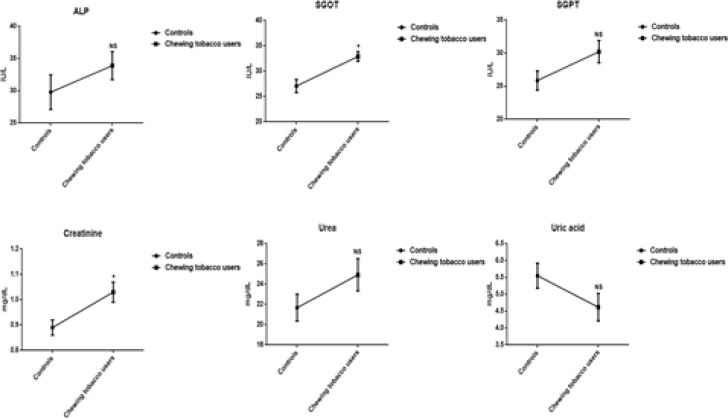
Concentrations of Liver Marker Enzymes and Kidney Markers in Plasma. Note: SGOT-Serum glutamate oxaloacetate transminase; SGPT-Serum glutamate pyruvate transminase; ALP-Alkaline phosphatase

**Table 1:** Plasma Biochemical Profile in Chewing Tobacco Users

Parameter	Controls	Chewing tobacco users	P value
Glucose (mg/dl)	86.38±3.08	92.08±3.64NS	0.24
Albumins (g/dl)	3.97±0.15	3.64±0.16 NS	0.15
Globulins (mg/dl)	2.78±0.15	2.25±0.24 NS	0.078
Total proteins (mg/dl)	6.40±0.20	6.78±0.25 NS	0.25
Iron (mg/dl)	121.40±2.30	109.45±4.06*	0.01
Hemoglobin (g/dl)	14.26±0.92	9.96±0.45*	0.0004
Glycolipids (mg/dl)	269.42±7.13	218.85±6.01*	≤ 0.005
Amino acids (mg/dl)	5.03±0.32	3.74±0.13*	0.001

NS, Not significant

**Table 2:** Correlation of Nicotine and Cotinine in Plasma, Saliva, and Urine of SLT Users

	Saliva nicotine		Saliva cotinine		Urine nicotine		Urine cotinine	
	r	P	r	P	r	P	r	P
Plasma nicotine	-0.42	0.16			0.38	0.22		
Plasma cotinine			-0.041	0.89			0.046	0.88

r, Correlation coefficient; P ≤0.05.

**Figure 3c:**
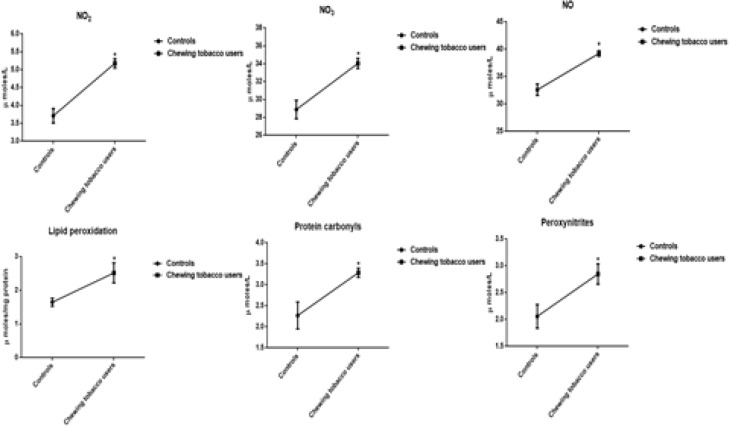
Levels of Nitroxidative Stress Markers in Chewing Tobacco Users. Note: NO-Nitric oxide; NO_2_-Nitrites; NO_3_-Nitrates

**Figure 4:**
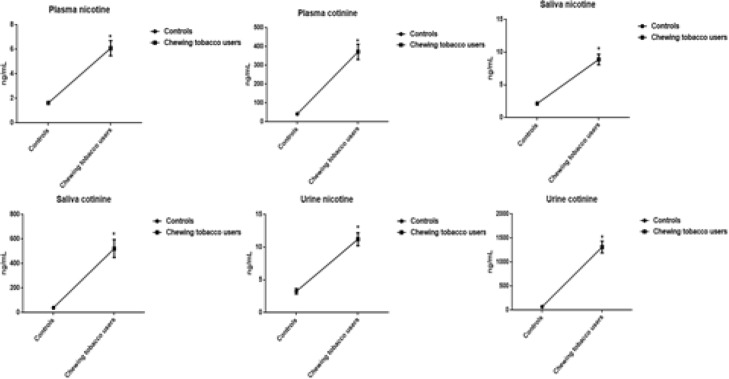
Concentrations of Nicotine and Cotinine in Plasma, Saliva, and Urine

**Figure 5a:**
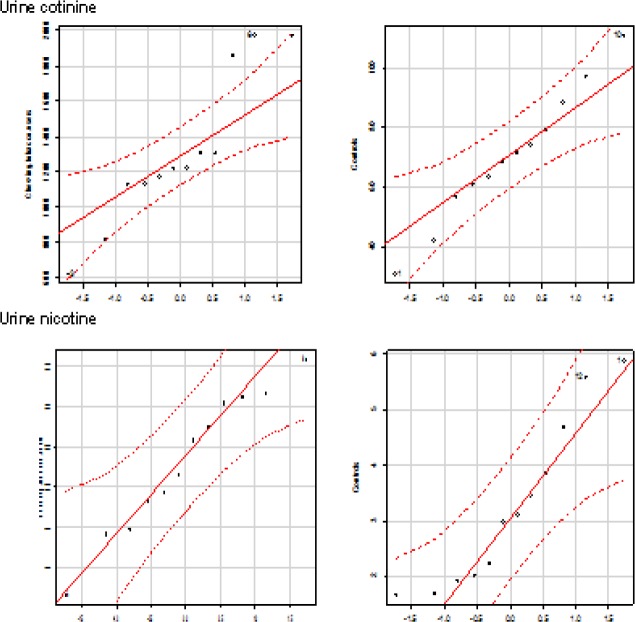
Quantile Comparison Plots of Cotinine and Nicotine in Urine

**Figure 5b:**
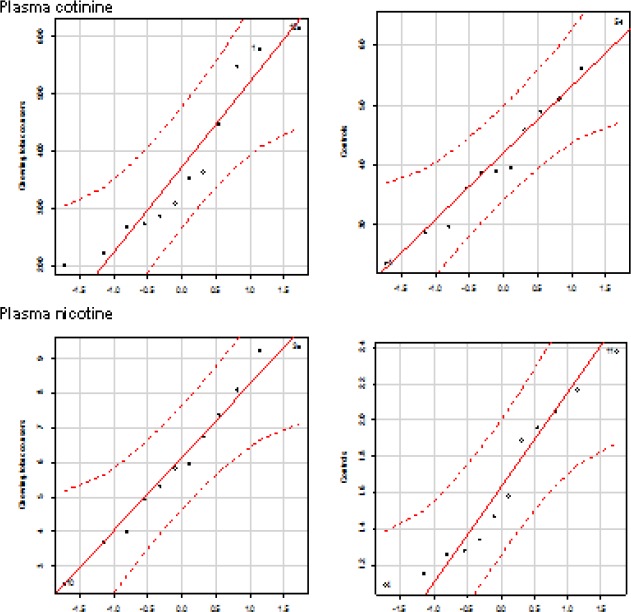
Quantile comparison plots of cotinine and Nicotine in Plasma

**Figure 5c:**
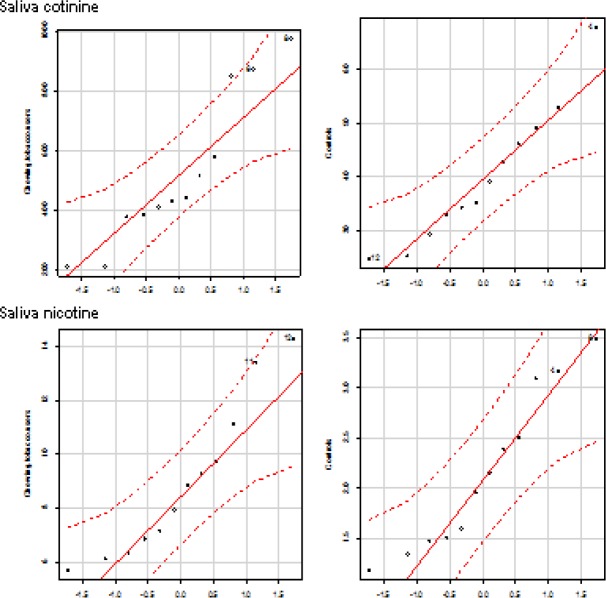
Quantile Comparison Plots of Cotinine and Nicotine in Saliva

## Discussion

Our study demonstrated that the significant increase in the levels of nicotine and its metabolite cotinine in plasma, saliva, and urine of chewing tobacco users. Previous reports stated that salivary concentrations of nicotine and cotinine observed in gutkha and khaini users (Begum et al., 2018). During the 2-year study, increase in the plasma nicotine and cotinine levels was detected in male and female Wistar Han rats exposed to similar concentrations as same as human exposure (Theophilus et al., 2015). Nicotine is able to cause an increased risk of cardiovascular, respiratory, gastrointestinal disorders, decreased immune response and disturbances on the reproductive health. It affects the cell proliferation, oxidative stress, apoptosis, and DNA mutation by various mechanisms which lead to cancer (Mishra et al., 2015). Nicotine is the major component in all tobacco products that has a major role in the development of dependence and addiction (Siqueira et al., 2017).

There is a direct association between salivary cotinine and the severity of periodontitis can be reported (Surya et al., 2012). The strong link exists between the serum cotinine concentration and tobacco smoke-induced emphysema in mice (Xu et al., 2014). The urinary levels of cotinine by HPLC-MS/MS in a population to assess their exposure to active and passive smoking (Paci et al., 2018). The cotinine acts as major metabolite and have great potential to become effective agents to prevent and alleviate neurological symptoms developed in subjects with Parkinsonism (Barreto et al., 2015). Nicotine and cotinine inhibit catalase and glutathione reductase activity, contributing to an accumulation of reactive oxygen species (ROS) by cigarette smoke exposure (Aspera-Werz et al., 2018). The smokeless tobacco users have increased risk of both heart disease and stroke in US compared to Swedish smokeless tobacco users (Rostron et al., 2018).

The experimental studies are helpful to develop the relationship between smokeless tobacco and disease pathology like immune, cardiovascular, and reproductive dysfunctions (Willis et al., 2012). The treatment of smokeless tobacco extract induced higher concentrations of reactive oxygen species and malondialdehyde in cultured human oral mucosa fibroblasts cells (Li et al., 2018). Peroxynitrite, though not a free radical by chemical nature, is a powerful oxidant that reacts with proteins, DNA, lipids, oxidative injury, committing cells to necrosis or apoptosis (Islam et al., 2015). The levels of peroxynitrites are increased in plasma of chewing tobacco users. Peroxynitrites are actively involved in the processes of protein carbonylation and lipid peroxidation and act as endogenous cytotoxin in pathological disease conditions (Radi, 2018). The reactive oxygen-nitrogen species (RONS) like nitric oxide, peroxynitrites, protein carbonyls, and malondialdehyde are significantly higher in the plasma. 

The active ingredient of smokeless tobacco is nicotine has been energetically engaged to enhance the levels of malondialdehyde in plasma of experimental subjects. The lipid peroxidation products are highly reactive and display marked biological effects, which, depending upon their concentration, cause selective alterations in cell signalling, protein and DNA damage, and cytotoxicity (Ramana et al., 2013). Our results manifested that smokeless tobacco users establish that the significant increase in the concentrations of nitric oxide (Nitrites and nitrates) of plasma. Nitric oxide could be able to dilate the blood vessels and improve the circulation at low concentrations and at high concentrations, it can cause circulatory shock and induce cell death (Achike and Kwan, 2003). NO has been convey to induce process of nitrosylation and nitrotyrosination in the protein modifications, organelle fragmentation, and inhibit mitochondrial respiratory complexes (Knott et al., 2009). Plasma profile including fasting glucose, kidney markers (creatinine and urea), liver marker enzymes (SGOT, SGPT, and ALP), lipid profile (cholesterol, triglycerides, LDL-C, and HDL-C) have distinguished in the normal range of plasma in chewing tobacco users compared to normal controls. 

In conclusion, chewing tobacco users revealed a significant increase in levels of nicotine and cotinine in plasma, saliva, and urine. The significantly higher levels of nitric oxide, peroxynitrites, protein carbonyls, and malondialdehyde act as indicators of development of oxidative stress in response to nicotine and tobacco-specific N-nitrosamines. The chewing tobacco users communicated the normal range of plasma biochemical profiles compared to other smokeless tobacco products (Panmasala with tobacco, gutkha, and khaini).
